# Decision-maker led implementation research on immunization: learning from low- and middle-income countries

**DOI:** 10.1186/s12961-021-00720-2

**Published:** 2021-08-11

**Authors:** Zubin Cyrus Shroff, Arielle Buijs Mancuso, Alyssa Sharkey, A. S. M. Shahabuddin, Binay Kumar, Hope Johnson, Abdul Ghaffar

**Affiliations:** 1 Alliance for Health Policy and Systems Research, WHO, Geneva, Switzerland; 2 Implementation Research and Delivery Science Unit, Health Section, UNICEF, New York, USA; 3 Gavi, the Vaccine Alliance, Geneva, Switzerland

The past decade has witnessed a significantly increased interest in implementation research, a field of health policy and systems research (HPSR) defined as the “scientific inquiry into questions concerning implementation—the act of carrying an intention into effect, which in health research can be policies, programmes, or individual practices (collectively called interventions)” [1]. Among other factors, this was spurred by a growing realization that the ineffective implementation and scale-up of proven interventions hindered the achievement of the health-related Millennium Development Goals in many countries, a reality that persists in the Sustainable Development Goals era [2]. Immunization is an exemplar of such a proven, highly cost-effective intervention that averts up to three million deaths annually and is responsible for a reduction of more than 80% in measles incidence since the year 2000 [3, 4]. Despite this, in 2018, nearly 20 million infants did not receive the third dose of the diphtheria, pertussis and tetanus vaccine, a widely used marker for effective vaccination coverage. Sixty percent of these infants resided in 10 low- and middle-income countries (LMICs) [5].

Given the focus of implementation research on enhancing the implementation of interventions (including programmes, policies and individual practices), the extent and process of engagement of stakeholders beyond researchers in implementation research has emerged as a critical focus over time. Implementers have moved from more traditionally conceptualized roles as passive “knowledge users” to being active coproducers with researchers in the identification of research questions and generation of new scientific insights and knowledge products [6, 7].

The involvement of these decision-makers in identifying and developing research questions is thought to increase the likelihood that what is being researched will address their concerns and be of relevance and use to the health system [7, 8]. This collaboration may also then facilitate the uptake of research findings, including through their integration into decision-making processes. In addition, decision-maker engagement in the research process can facilitate access to “insider” perspectives and knowledge including tacit knowledge, allow for greater research ownership and acceptability, and create safe spaces to discuss difficult findings, with a focus on improvement [9]. All of these factors are essential to bring about the embedding of research into policy and decision-making, a situation “where researchers and decision-makers are linked through a system in which the need for evidence to inform policy is understood by decision-makers” [10].

Empowering decision-makers to play a central role in the research process is intrinsic to bringing about the embedding of research described above [8, 9]. One strategy towards bringing about the needed power shift is to engage decision-makers as principal investigators of the research, something that is central to the initiative described below.

Beginning in 2015, the Alliance for Health Policy and Systems Research (AHPSR) within WHO and the United Nations Children’s Fund (UNICEF) in partnership with Gavi, the Vaccine Alliance (GAVI), began supporting a programme of decision-maker led research focused on immunization (Decision-Maker Led Implementation Research on Immunization, or DELIR). This was informed by (a) recognition of the importance of immunization as an intervention whose implementation and scale-up is critical to move towards universal health coverage, (b) a felt need for new knowledge to better understand and help overcome health systems barriers to effective scale-up and implementation of immunization interventions, and (c) recognition of the added value of engaging decision-makers as principal investigators through an embedded approach informing the generation, dissemination and use of this new knowledge. The overall purpose of the DELIR initiative was to support the generation of new knowledge to inform the implementation of immunization interventions with the aim of improving coverage in LMICs.

Two calls for research were issued in 2015 and 2016 targeted at selected countries with high child mortality and/or low immunization coverage (Table 1), and responding to identified priority issues around the implementation of immunization programmes (Table 2). To be eligible for funding, the principal investigator of the research had to be a decision-maker, defined as an individual directly involved in the implementation of an immunization programme or service within the health system in an eligible country. This included programme managers, district health officers, nongovernmental providers, public or private practitioners and front-line health workers. The research team was also required to include at least one researcher affiliated with an academic or research institution based in the study country.

**Table 1:** List of eligible countries

Afghanistan	Indonesia	Pakistan
Central African Republic	Kenya	Papua New Guinea
Chad	Madagascar	Somalia
Democratic Republic of the Congo	Mozambique	South Sudan
Ethiopia	Myanmar	Uganda
Haiti	Niger	Yemen
India	Nigeria

**Table 2:** Priority issues supported under research programme

Identifying caregivers’ barriers to immunization services in urban slum areas—Pakistan
Adapting and testing strategies or tools to assess the effectiveness of demand-creation communication—Chad, India
Health and immunization systems—Ethiopia
Demand and vaccine hesitancy—Nigeria
Programme management, monitoring and evaluation strategies—Democratic Republic of the Congo, Uganda
Vaccination and coverage—Nigeria

In total, 125 letters of intent were received in response to the calls for research; 46 of these teams were invited to submit full proposals. After a process of independent review, a total of 14 research projects in 10 LMICs in Africa and Asia were supported under this programme (Table 3). Criteria that determined selection included (a) potential for the research to make a difference in the delivery of an immunization programme or service, (b) value for money, (c) and institutional capacity to conduct the research, as well as (d) ensuring diversity in terms of issues addressed by the research. Research grants of up to a maximum of US$ 100,000 were provided for a period of 12 months.

**Table 3:** List of selected projects and priority issues addressed

Country	Title	Priority issue	Level of study
Chad	More responsive immunization services through tailoring for hard-to-reach populations in Chad	Adapting and testing strategies or tools to assess the effectiveness of demand-creation communication	District
Democratic Republic of the Congo	Strengthening health information systems in support of national vaccination programs in the Democratic Republic of the Congo	Programme management, monitoring and evaluation strategies	National
Ethiopia	How can the use of data within the immunization program be increased in order to improve data quality and ensure greater accountability?	Health and immunization systems	Regional
Ethiopia	Lack of functional linkages and feedback mechanisms among different health facilities along with mobility of caregivers affects follow-up visits in utilizing the routine immunization services	Health and immunization systems	Sub-district (sub-city)
India	Negative social media messages on vaccines: How can the resultant trust deficit between caregivers and health workers be overcome? A qualitative enquiry in Malappuram district of Kerala state in India	Adapting and testing strategies or tools to assess the effectiveness of demand-creation communication	District
Kenya	Emerging hesitancy upon new vaccine introduction: tackling a most unusual barrier	Demand and vaccine hesitancy	Sub-district (facility)
Nigeria	Increasing the utilization of immunization in Ogun state of Nigeria using participatory evaluation and action research	Vaccination and coverage	Sub-state (local government area)
Nigeria	Potential role of civil society organization engagement for increasing the demand for and uptake of immunization services in Odukpani Local Government Area of Cross River state of Nigeria	Demand and vaccine hesitancy	Sub-state (local government area)
Nigeria	Use of social actors to address contextual barriers for utilization of immunization services among caregivers of under-five children in urban slums of Yobe state, Nigeria in the context of Boko Haram insurgency	Identifying caregivers’ barriers to immunization services in urban slum areas	Sub-state (urban slums)
Pakistan	Improving vaccine uptake in urban slums of Karachi, Pakistan—implementation research to explore and address supply- and demand-side barriers to routine immunization	Identifying caregivers’ barriers to immunization services in urban slum areas	Sub-district (urban slums)
Somalia	Examination of ministry of health engagement barriers in demand-generation strategies and application of improved engagement in developing institutional capacity to increase vaccine immunization uptake in Puntland	Health and immunization systems	State
Uganda	Process evaluation of community health facility-based microplan development and implementation in two districts in Uganda	Programme management, monitoring and evaluation strategies	District
Uganda	Evaluating the role of leadership in transitioning vertical into integrated and sustainable district health programs—a case study of immunization in Luuka district, Uganda	Health and immunization systems	District
Viet Nam	Governance of immunization program for children 0–23 months in Viet Nam	Health and immunization systems	Provincial

Teams were provided ongoing technical support by the AHPSR and UNICEF in the form of regular feedback on project deliverables as well as more intensive support through in-person workshops focused on protocol development and data analysis and dissemination, respectively. Workshops lasted for 5 days each. The protocol development workshops provided an overview of implementation research to participants before going into group work. The latter entailed intensive engagement between facilitators and research teams towards the identification of study goals and objectives, and the development of research questions and data collection tools, with teams receiving extensive feedback from both facilitators and other research teams through plenary sessions. By contrast, the data analysis workshop focused on developing recommendations and products targeted at decision-maker audiences. This included engagement with facilitators around how to interpret research findings towards developing recommendations for policy-making, sensitization to relevant methods such as stakeholder analysis, and support in developing a dissemination strategy. All workshops were jointly facilitated by representatives from AHPSR and UNICEF. Projects were completed between 2015 and 2018.

This special issue of *Health Research Policy and Systems* brings together findings from eight of these projects conducted in six countries (Chad, Ethiopia, India, Nigeria, Pakistan and Uganda). In addition to representation of a diverse group of countries across Africa and Asia, the collection brings to the fore the use of diverse methods and approaches. While the majority of the studies (7 out of 8) use mixed methods bringing together key informant interviews, focus group discussions and document reviews, the collection includes examples of innovative approaches such as participatory action research towards understanding how interventions to increasing the utilization of immunization in Nigeria have met their objectives. Published papers represent the diversity of priority issues supported under the larger research programme, with two papers each speaking to issues around immunization systems (both papers from Ethiopia), assessing the effectiveness of demand creation communication efforts (Chad and India), and programme management, monitoring and evaluation strategies (Uganda). One study each addresses issues around immunization challenges in urban slums (Pakistan), demand and vaccine hesitancy (Nigeria), and vaccination and coverage (also from Nigeria). Responding to each of the six thematic areas identified in the original calls for research, studies were conducted at the sub-district (Ethiopia and Pakistan) as well as district (Chad, India and Uganda), sub-state (both papers from Nigeria) and regional levels (Ethiopia). The issue concludes with a synthesis paper that systematically analyses experiences and perceptions of those involved in the projects and draws lessons for the further development of decision-maker led strategies going forward.

The overwhelming response to the calls for research demonstrates a high level of decision-maker interest in generating and using research to strengthen implementation, something that bodes well for evidence-informed decision-making. The extent of decision-maker involvement throughout the projects, including at protocol and analysis workshops, also highlighted the feasibility of the decision-maker led approach, contrary to the way in which research is traditionally carried out. Third, despite short timelines, several projects including those in Nigeria, Chad and Ethiopia were able to demonstrate a contribution to change at the level of programme and policy implementation. Finally, several projects also discussed ongoing activities building on the research findings to strengthen implementation demonstrating a catalytic value of this approach to research [11].

We believe that this research programme, led by decision-makers and using the embedded approach to facilitate the incorporation of evidence into decision-making, is but a first step. By demonstrating the added value of incorporating evidence generation and use within the implementation of programmes, we hope to spur funders and ultimately national governments to invest in strengthening research capacity at both the individual and institutional levels. This in turn will be central towards enabling the realization of a long-term vision where the generation and use of research is a routine part of programme implementation, something that we argue is critical for stronger health systems.

## Conclusions

- Decision-maker led research is a promising approach to align health systems research to policy and decision-making priorities.
- This supplement issue of *Health Research Policy and Systems* brings together examples of decision-maker led research to address challenges around immunization in Asia and Africa.
- There is great interest in this approach based on the overwhelming response to calls for proposals issued.
- The papers in this supplement demonstrate the added value of incorporating evidence generation and use within programme implementation. This should spur funders and national governments to invest in strengthening individual and institutional research capacity.

## Data Availability

Not applicable. The manuscript does not contain any data.
